# Chicago Gynæcological Society, February 9th

**Published:** 1886-04

**Authors:** 

**Affiliations:** 2330 Indiana avenue


### SOCIETY REPORTS.

TRANSACTIONS OF THE CHICAGO GYNÆCOLOGICAL SOCIETY.

*LXIII Meeting, Friday, February 19th, 1886.*

I.—JACKSON. *Laparotomy for Pelvic Abscess.*

II.—WAXHAM. *Description of a Feeding Bottle for Use in Cases of Intubation of the Larynx.*

The PRESIDENT, DANIEL T. NELSON, M. D., in the chair.

I. PROFESSOR A. REEVES JACKSON, M.D., opened the adjourned discussion of the treatment of pelvic abscess by the presentation of the following paper, entitled,

LAPAROTOMY FOR PELVIC ABSCESS.

Owing to Professor Jackson's absence, the paper was read by the SECRETARY, EDWARD WARREN SAWYER, M. D.

At the December meeting of this SOCIETY, a discussion arose upon the subject of pelvic abscess and its treatment. It was based upon the report of a case by Dr. H. T. Byford, which had been treated by favoring discharge of the pus by way of the rectum, and the placing within the abscess cavity a portion of sulphate of copper to promote granulation.

The discussion seemed unfinished, and was withal of so interesting a character, that I have thought well to reopen it by the relation of the following case. Whether the operation performed in the treatment of the case should be termed a laparotomy will depend upon what significance we attach to the term. Upon this point opinions will doubtless differ.

If we understand by the word laparotomy, the opening of the abdominal cavity in its largest sense, the term is here correctly used; but if, on the other hand, we mean the opening of the abdomen for the relief of *an encysted intraperitoneal abscess non-adherent to the abdominal wall*, the term would not be properly applicable, for here there was complete adhesion, and, possibly, in the course of time the abscess might have pointed and opened in that direction, although I do not believe that such result would have occurred. Without active interference I think the disease would have resulted in death from pyæmia in a very few days.

On March 9, 1885, I visited Anna N., in consultation with Dr. Louis Braun, of this city. She was twenty-four years old, had been married six years, and had one child eighteen months old. On February 1—five and a half weeks prior to my visit—she had miscarried, producing a fœtus four months old. A few days after that event, Dr. Braun found the patient suffering from symptoms of pelvic inflammation, which had since continued, with varying severity. He informed me that the pelvic swelling, which he detected on examination, appeared to involve all the periuterine structures, but to a greater extent on the right side; that during the past few days, however, it had seemed greater on the left side. From the onset of the attack the pulse had been rapid, and the temperature elevated—the former ranging from 110 to 130, and the latter being persistently over 102° F., reaching, on one occasion, 104° F. Pain had been severe, but controllable by morphia. The appetite had failed utterly and the stomach finally rejected all food.

At the time of my visit, the patient was pale, extremely emaciated, and her visage showed marks of prolonged suffering.

On examination, I found on the left side of and behind the uterus, a swelling as large as a medium-sized orange, with rather indistinct outlines. Its lower portion was in a plane with, or somewhat below, the *os uteri*, and bimanually its upper margin could be felt extending above the fundus, which was pushed strongly to the right. Both uterus and tumor were immovable. The latter had a slightly elastic feeling in some places, although I was unable to detect any certain fluctuation through the vagina, rectum or hypogastrium. Through the posterior vaginal wall, at a point about an inch above the lower portion of the swelling, I fancied I received a sensation of bogginess, and this, taken in connection with the history of the case, gave me the belief that pus was present. Accordingly, I thrust a curved trocar and canula into the swelling by way of the vagina to the depth of about two inches, with no other result than the emission of a few drops of blood.

It was then concluded that the patient should have prolonged hot water vaginal douches daily, rectal feeding, and appropriate anodyne and tonic medicines.

On April 18,—five weeks later,—I saw the patient again, with Dr. Braun, who reported that after my former visit the symptoms had all become gradually ameliorated; the stomach resumed its functions, pain subsided, pulse and temperature became normal. No menstrual or other discharge had appeared. This improved condition had continued until two days before, when, without apparent cause, the patient had a chill, followed by rapid pulse, high temperature, pelvic pain and irritability of the bladder.

Under anæsthesia I examined the abdominal and pelvic organs. The pelvic swelling had undergone no marked change, except that it seemed to have increased in an upward direction, extending now to a point about an inch above the *symphysis pubis*. At this place I thought I detected obscure fluctuation. The swelling as felt *per vaginam* was hard at every accessible point. All operative measures were declined by the patient and her friends, and the treatment advised consisted in the administration of morphia and quinine, and peptonized milk for diet.

*April 19.*—The patient was much worse. The pelvic pain was controlled only by large doses of morphia given hypodermically, and the stomach retained almost nothing. The pulse was 130, temperature 102° F. It was decided that laparotomy should be performed the following day.

*April 20.*—There were present as assistants Doctors Steele, Braun, Sterl, Dickerson and Mascheck. A spray of carbolized water had been kept playing in the room for several hours. The patient was etherized, and the bladder emptied by catheter. She was the thinnest person I ever saw placed upon an operating table. Immediately before the taking of ether her pulse was 124, temperature, 103° F.

The hair of the pubis was shaven off, and the skin of the abdomen washed with soap and carbolized water. An incision three inches long, ending below at the upper portion of the *mons veneris*, was made in the middle line of the hypogastrium. Deepening the cut, I came upon the peritoneum, which, however, could not be separated from the parts beneath. Proceeding inward through dense structures the knife suddenly entered an abscess cavity, which at once gave exit to a stream of pus to the amount of two or three ounces. Passing my finger through the opening, I found that the cavity extended downward, behind and to the left of the uterus, about three inches. The abscess walls proper could not be accurately defined. The inflammatory processes had matted together the upper part of the uterus, the left broad ligament, tube and ovary. The cavity was washed out, and a rubber drainage tube passed to the lower end, the outer portion the tube being stitched to the edge of the wound at its lower extremity. The remainder of the wound was closed with sutures, and dressed in the usual manner.

The night following the operation the patient slept fairly well without an opiate.

When I saw her the next day she had taken milk and lime water with relish; her pulse was 108, and temperature $100\frac{2}{5}°$ F.

In brief, the relief of the symptoms was immediate, and the recovery uninterrupted. Pus continued to discharge for more than six weeks in constantly diminishing quantity. The tube was then removed. Menstruation appeared July 20th, and has been regular since. I examined the patient September 25th. The uterus was still in a position of right lateroversion, but movable in a slight degree. The parts about the left broad ligament were thickened, and somewhat tender. An irregularly shaped mass occupied Douglas's space, and extended upward and to the left. The patient had gained greatly in weight, was ruddy, and doing her own housework. In a letter dated December 29th, she states, "I have no pain, and feel better than I have for four or five years."

PROFESSOR JACKSON appended the following note from MR. LAWSON TAIT:

7 THE CRESCENT,
BIRMINGHAM, Jan. 4th, 1886.

*My Dear Sir:* I have performed now thirty-two operations for pelvic abscess, in every one of which a cure has resulted.

Yours very truly
LAWSON TAIT.

DISCUSSION.

PROFESSOR CHRISTIAN FENGER: Before entering into the discussion of the paper which has been read here this evening, I wish to remark that I came here under the impression that the entire subject of suppurative, pelvic inflammation was to be dealt with ; I now see the subject is limited to the treatment of pelvic abscess by laparotomy. The operation performed in Dr. Jackson's case I should not call a laparotomy at all, but simply an oncotomy. An abscess was opened, and the operation does not differ materially from the opening of a deep-seated abscess in any other region of the body—*e. g.*, in an extremity. As I understand the term laparotomy, and I am not aware that it is ever used otherwise, it means that section of the abdominal parietes is followed by an operation, performed within the peritoneal cavity. If the wall of an abscess situated in an abdominal organ has become adherent to the visceral surface of the abdominal parietes, the peritoneal cavity is of necessity obliterated to the extent to which adhesions have formed. An incision made over such adhesions does not open the peritoneal cavity, and consequently the operation cannot be spoken of as a laparotomy. In the paper which I published on "Laparotomy for Periuterine Abscess," it was distinctly stated that the only way by which it seemed possible to get at the abscess was by opening the peritoneal cavity; it is also mentioned that omentum and intestines were found between the walls of the abscess and the walls of the abdomen.

Concerning the etiology of pelvic abscess, I should like to call attention to the literature of the subject. Sänger,[1] whose statements regarding etiology I have found to be the most complete, says, that one out of nine of all gynæcological affections is of gonorrhœic character. He further says that fifty per centum of these are diseases of the uterine appendages ; although, of course, any part of the genital tract may be primarily invaded. In the Fallopian tubes, he finds that disease most often has its principal focus, where it begins and whence it spreads. He distinguishes six kinds of salpingitis: (1), septic, puerperal and non-puerperal; (2), tuberculous; (3), syphilitic; (4), actinomycotic; (5), gonorrhœic; (6), a mixed form. The gonorrhœic is the most common form of the disease, and it produces the most severe cases of pelvic inflammation.

It has not as yet been proven that the gonococci of Neisser can, of themselves, produce abscesses; but destruction of the surface of the mucous membrane is sufficient; an entrance is thus given to the septic, pus microbes, the *staphylococcus aureus* and *albus* and the *streptococcus pyogenes*, which are probably always present.

The invasion having taken place, we must ask ourselves by what channel does the inflammation travel? Where should we expect finally to find an abscess in case one should form? The Fellows will remember the beautiful experiments of Bitas, Koenig,[2] Schlesinger[3]; experiments which about three years ago I repeated in the dead-house of the Cook County Hospital, although the purpose I had in view at that time was a different one. These gentlemen injected, by means of fine canulæ, fluids, such as colored glue, into the periuterine tissues of puerperal and non-puerperal bodies. Koenig found (*a*) that fluids injected in the region around the *fundus uteri* and uterine portion of the Fallopian tubes, first pass upwards into the iliac fossa to reach the crest of the ilium, then downwards towards Poupart's ligament, and finally into the *pelvis minor* or true pelvis; (*b*) fluids injected into the periuterine tissues, in the neighborhood of the internal os, first fill the extraperitoneal connective tissue of the *pelvis minor*, then follow the round ligament as far as Poupart's ligament and ascend in a backward direction into the iliac fossa; (*c*) that when the injection is made near the lower portion of the posterior surface of the uterus, the fluid first flows into the *cul de sac* of Douglas and thence rises into the iliac fossa.

Schlesinger, although in the main agreeing with Koenig, differs with him in the following two points: He says, (*a*) when fluid is injected into the neighborhood of the *fundus uteri*, it first passes into the iliac fossa, but thence it does not descend into the true pelvis, as Koenig observed, but it ascends, running up the anterior abdominal wall; (*b*) from the broad ligament the fluid finds its way into the iliac fossa and thence upwards towards the kidney, running in the mesentery of either the ascending or descending colon. Schlesinger further makes the interesting statement that his pericervical injections filled the pericervical tissues, but that they never produced a tumor which could be felt above the *symphysis pubis*.

As far as my experience goes, the results of these experiments correspond well with the clinical facts. The puerperal abscesses which I have opened were situated, two over the crest of the ilium, one on Poupart's ligament, and one on the anterior abdominal wall, about three inches above the ligament.

As before mentioned, about three years ago I made similar experiments; the fluid I employed was milk. My object, at the time, was to ascertain the exact relative position of such an artificial exudate, representing an abscess, with regard to the anterior wall of the abdomen, especially of an exudate in one of the broad ligaments. I wanted to see for myself what difficulties I must be prepared to encounter in uniting the walls of a pelvic abscess, after having opened it, to the edges of the abdominal wound. As might have been expected, I found the difficulties of the operation to vary partly with the size ot the exudate and partly with the degree of tension of the abdominal parietes. On the whole, the matter seemed simpler to me than I had *a priori* imagined.

Whether in cases of pelvic inflammations and abscesses laparotomy should be done or not, is a question of comparatively recent date, it being but little older than five years. As I have already said in my paper on "Periuterine Abscess," the operation is always to be regarded as a last resort and should never be thought of in cases in which the abscess can with safety be reached in any other way, which, of course, excludes opening it through the rectum. Lawson Tait,[4] of Birmingham, and Martin, of Berlin, were the first who attempted to prevent the terrible contingencies of pelvic inflammations by attacking the disease at its original seat; Lawson Tait[5] removed the suppurating uterine appendages, Martin[5] operated for suppurating, periuterine hæmatocele. Tait operated for a suppurating hæmatoma of the right Fallopian tube (peritonitis), in 1878, and he removed both tubes for pyosalpinx and an ovary for abscess in 1881. In 1881 Martin[6] performed laparotomy in three cases of intraperitoneal hæmatoma, *i. e.*, retro-uterine hæmatocele. He opened the peritoneal cavity, incised the sac, and evacuated the blood and pus; he then drained into the vagina, through the pouch of Douglas, and closed the opening he had made into the sac from the peritoneal cavity by sutures. In the discussion following the reading of Martin's paper, Kaltenbach opposed Martin's operation, and pleaded for an extraperitoneal operation, reaching the abscess either from above Poupart's ligament, or, as Hegar recommended, from the ischio-rectal fossa. In 1880, Feldman,[7] of Goettingen, published an operation for double pyosalpinx. In 1882, Baumgaertner published a case of hæmatocele in which Martin's operation had been successfully performed. These more or less sporadic operations called the attention of the profession to the subject, and already during the following year, 1883, upwards of fifty or sixty cases were reported, in which laparotomy was resorted to for the cure of pelvic inflammations. Aside from the forty-six cases which Lawson Tait published in his book, "Diseases of the Ovaries," he reported seven[8,9] more. T. Gaillard Thomas[10] reported five cases; Zeiss,[11] Thornton,[12] Baer,[13] and Proschownick,[14] each, one. In 1884, America was represented by fifteen cases; Stone,[15] 1; Lee,[16] 4; Lusk,[17] 1; Martin,[19] of Chicago, 1; Goodell,[20] 2; Jones,[21] 1; Thomas,[22] 1; Dawson,[23] 1; Polk,[24] 3. In England we have thirty-five cases; Tait,[25] 15, 7 of pelvic abscess; McDonald,[28] 2; Lediard,[29] 1: Chapman,[30] 1; Savage,[26] 9, 8 of pelvic peritonitis and 1 of hæmatocele; Malin,[27] 7.

In Germany, twenty-two cases were reported: Martin,[31] 8 of suppurating hæmatocele; Gusserow,[32] 7, in 4 of which the sac was stitched to the abdominal wound; Saenger,[33] 5; Schroeder,[34] 1; Quetsch,[18] 1.

It is evident that the operation rapidly gained ground, and that laparotomy has come to occupy a prominent place in the treatment of pelvic inflammations and abscesses. It may be objected that as yet the indications for the operation are not as clearly defined as we might wish them to be; in answer to this, we can only say that the operation is new and that we must consider the importance of the subject the guaranty of progress in the right direction.

I wish to add a few words about the subject, as it is modified, in cases in which the periuterine abscess communicates with the rectum or some other part of the intestines. My remarks will have reference to the discussion of my paper on "Chronic Periuterine Abscess," the discussion of to-night being but a continuation of that broken off at our recent meeting for want of time. My paper was read before this Society in May, 1885. I will refer as well to the discussion of Dr. H. T. Byford's paper on "Pelvic Abscess," read here December 18, 1885.

When a communication exists between an abscess and the intestinal tract, evacuation of the pus into the bowel is sometimes followed by spontaneous recovery. As a rule, however, such a condition is extremely dangerous, for the abscess cavity is constantly being infected with septic material from the intestine. Péan in his *Diagnostique et Traitement des Tumeurs de L'abdomen et du Bassin*, T. 11, p. 155, writes that a periuterine abscess may open into the cœcum, colon or rectum; that if a periuterine abscess opens into the intestine, bladder or uterus, septic infection will not fail to produce its symptoms and may speedily prove fatal. This statement may be a little too broad. Schroeder, in his *Krankheiten der Weiblichen Geschlechtsorgane*, Leipzig, 1880, says that when the abscess has broken into the gut or bladder we have to deal with a grave condition, as it is difficult or impossible to reach the abscess from the vagina. At that time, in 1880, he had not thought of the practicability of laparotomy in such cases. When the abscess has evacuated itself into the rectum, he considers it less difficult to get access to it, and he advises cutting through the posterior *cul de sac* of the vagina, and dissecting upwards between the uterus and the rectum. Emmet[39] writes that when an abscess opens into the rectum, the case is very much complicated by septic infection through the fæces. The quotations given are sufficient for us to conclude that, when there is communication between a periuterine abscess and the rectum, the patient is in great and constant danger of dying from septic infection, and that, therefore, an operation should be done if possible to provide free drainage of the abscess. In discussing my paper, Professor W. H. Byford said that he was opposed to the line of treatment suggested by me, *i. e.*, laparotomy; that the *sphincter ani* should be dilated or incised, the communication between the abscess cavity and rectum should be dilated with the finger, a steel dilator or the knife, the cavity of the abscess scraped, washed out, and drainage effected *per rectum*

Aside from the old, now justly abandoned, puncture of a retrouterine abscess *per rectum*, Professor Byford's method of attacking such an abscess is to me entirely new. I have nowhere, in the course of my reading, met with a similar suggestion. If the abscess communicates with the intestine at a point beyond the rectum, a rectal operation is of course out of the question. If the opening into the rectum lies three inches or more above the anus, dilatation is hardly practicable. Such openings are often narrow and tortuous, the neighboring organs are immovable, and even if we divide the sphincter and the retrorectal tissues, we are obliged to work in the dark, for it is difficult or impossible to draw such an opening well down into view. The gentlemen present, who have extirpated a carcinoma of the rectum, will appreciate the difficulties of operating high up in the gut.

But above all, I must earnestly warn you against adopting Professor Byford's plan of employing in this region a knife for the purpose of dilating the opening of an abscess. Working here with a knife, we always run the risk of opening the peritoneal cavity and of dividing large vessels which are found in the wall of the sac. As I once demonstrated in the walls of an abscess, situated in a broad ligament. The large uterine vessels may be found anywhere, and if wounded, it is next to impossible to ligature them securely.

In a relatively small number of cases the abscess breaks into the rectum near the anus; in these, dilatation may be tried, and it may even effect a cure, as we learn from Dr. H. T. Byford's case. However, we must have heard of more than one case before we can judge of the value of this method of treatment. Being somewhat enthusiastic over his rectal method, Dr. Byford disposes of laparotomy by saying, "The treatment by abdominal section cannot for a moment be entertained, for at least two reasons," of which the first one is that it is necessarily followed by a recto-abdominal fistula, which is incapable of being promptly cured, and is apt to become an unfailing source of systemic infection. Of the numerous examples we have, I need but mention the peri-typhlitic abscesses to show that an intestino-abdominal fistula does not contraindicate the evacuation of the abscess. We operate to save life, whatever may become of the fistula afterwards; besides, these fistulæ do frequently close. In the third case referred to in my paper, it closed in two months. The closure of a cœcal fistula is a common occurrence. The explanation is not far to seek. Whenever an abscess breaks into the gut, the condition of the abscess necessitates such an outlet for the pus. When the abscess is drained through a counter-opening in the abdominal wall or the vagina, and thus transformed into a fistula, the necessity for emptying its contents into the bowel no longer exists, the opening has become useless and it gradually contracts.

A fistula in itself is no source of infection. We never hear for example, of a primary local tuberculosis originating in a fistulous tract. An anal fistula, when tuberculous, is always preceded by a local tuberculosis.

Dr. Byford, further on, criticises my cases in particular, remarking that to operate as does Lawson Tait, before the abscess has discharged, and then treat antiseptically, is an entirely different matter. All I have to say to this is, that the Doctor has entirely misunderstood Lawson Tait. A letter I had the pleasure of receiving from Mr. Tait one month after I had published my cases, begins, "I have just read your interesting articles in the *Annals of Surgery*." I shall now pass the letter around to the Fellows of the Society. In his second letter to me, Mr. Tait says, "Concerning your operations for pelvic abscess, I quite agree with you. Opening the abdomen is a very much more satisfactory way of dealing with these cases than any other." Dr. H. T. Byford's further remarks on my cases I shall pass by in silence, as I do not think their discussion is in order before the Chicago Gynæcological Society.

LITERATURE.

1. SANGER. *Centralblatt fur Gynæcologie, N. 41, 1884, p. 650.*

2. KOENIG. *Ueber die Bedeutung der Spaltraume des Bindegewebes.* Volkmann klin. Vortræge, N. 57. Chirurgie.

3. SCHLESINGER. *Œsterreichischer Med. Jahrbucher*, Heft 1 und 2, 1878. Virchow-Hirsch, Jahresbericht fur 1878; Bd. II, p. 579.

4. LAWSON TAIT. *British Med. Journal*, June 9, 1878.

5. LAWSON TAIT. *British Med. Journal*, March 14, 1881.

6. MARTIN. *Archiv fur Gynæcologie*, 1881, p. 463, und 1882, p. 476; also Virchow-Hirsch, Jahresbericht fur 1882, Bd. II, page 531.

7. FELDMANN. *Diss. Gœttingen*, 1879. Virchow-Hirsch, Jahresbericht fur 1880, Bd. II, p. 567.

8. LAWSON TAIT. Transact. Obstetric. Society of London, May, 1883 Virchow-Hirsch, Jahresbericht fur 1883, Bd. II, p. 578.

9. LAWSON TAIT. Ibidum, November, 1883

10. GAILLARD THOMAS. *Philadelphia Med. and Surg. Reporter*, March 3, 1883.

11. ZEISS. *Centralblatt fur Gynœcologie*, N. 47, 1883.

12. THORNTON. Transact. Obstetr. Soc., London, June, 1883.

13. BAER. *Amer. Journal of Obstetrics*, July, 1883.

14. PROSCHOWNIK. *Deutsche Med. Wochenschrift*, N. 36, 37, 1883.

15. STONE. *Medical News*, Philadelphia, August 23, 1884.

16. LEE. *Amer. Journal of Obstetrics*, June, p. 463.

17. LUSK. " " " " April, p. 383.

18. QUÉTSCH. *Centralblatt fur Gynœcologie*, N. 19, 1884.

19. MARTIN. *Chicago Med. Journal and Examiner*, August, 1884.

20. GOODELL. *Amer. Journal of Obstetrics*, August, 1884, p. 858.

21. JONES. Ibid., Novbr., p. 1155.

22. THOMAS. Ibid.

23. DAWSON. Ibid.

24. POLK. Ibid., February.

25. LAWSON TAIT. Transact. Pathol. Soc., London, XXXIII, p. 202. Harveian Soc., *London Lancet*, March 10, p. 473. *Medical Times*, Septbr. 6, p. 318.

26. SAVAGE. *Brit. Med. Journal*, March 8, p. 453.

27. MALIN. Transact. Med. Soc., London, Oct., p. 228.

28. McDONALD. *Edinburgh Med. Journal*, August.

29. LEDIARD. *Lancet*, Septbr. 20, p. 493.

30. CHAPMANN. *Archives de Tocologie*, Novbr., p. 964.

31. MARTIN (Aurelius). *Archiv fur Gynæcologie*, Bd. 23, p. 414.

32. GUSSEROW. *Charite Annalen*, IX Jahr.

33. SANGER. *Centralblatt fur Gynæcologie*, N. 32.

34. SCHROEDER. Ibid., N. 4.

35. W. H. BYFORD. *Chicago Med. Journ. and Exam.*, June, 1885, p. 510.

36. H T. BYFORD. Ibidum, Jan., 1886, p. 7.

37. PEAN. *Diagnostique et Traitement des Tumeurs de L'abdomen et du Bassin*, T. II, p. 155.

38. SCHROEDER. Krankheiten der weiblichen Geschlechtsorgane, Leipzig, 1880.

39. EMMET Principles and Practice of Gynæcology, p. 2861.

40. L. c., 510.

41. L. c., p. 7.

42. L. c., p. 13.

PROFESSOR W. W. JAGGARD begged permission to submit an extract of a letter from Dr. Paul F. Mundé, received about the fifteenth of February. Dr. Mundé's large experience in the treatment of pelvic abscess was well known to the Fellows of the Society.

Dr. Mundé writes: "I do not agree with Professor Byford's rectal treatment, as a rule, and certainly have not in my quite extensive experience found true laparotomy required to evacuate and drain a pelvic abscess. I mean opening the peritoneal cavity.

"Neither has my experience been that of Dr. Dudley, who says that the mortality, from abscess opening into the rectum, is great. I have seen only one case that I thought must die, in which there was a spontaneous rectal perforation."

Dr. Henry T. Byford: A few years ago Sir James Y. Simpson invented the operation of division of the cervix for uterine flexures. Almost all gynæcologists began performing it, and in a short time had done more harm than good with it. Only a few years since, Dr. T. A. Emmet invented the operation of trachelorrhaphy.

While justly maintaining that it is an exceedingly valuable operation in proper cases, he has recently stated that it may have done more harm than good. A short time ago, Lawson Tait invented a method of treating pelvic abscess by laparotomy, and surgeons are resorting to it with a certain degree of success. The method is undoubtedly gaining favor, and bids fair to be employed with disastrous frequency, and possibly do more harm than good.

In endeavoring to fix the limits of the usefulness of laparotomy for pelvic abscess, we must go beyond the recorded experience of the laparotomists, for the records are not yet all in. I understand that Tait opens the abdominal cavity only in those cases which cannot be reached from below.

PROFESSOR FENGER remarked, parenthetically: He (Tait) does not operate through the rectum. He performs laparotomy when he cannot open through the vagina.

DR. BYFORD, continuing: Then we may say that abscesses like the one reported (which point above) should be opened by a simple incision, without entering the abdominal cavity; and those which point in the vagina should be opened and treated through the vagina. Now the question is, should those which point in the rectum be opened and treated through the rectum? What I claim is, that the procedure has not been thoroughly tested by the profession. As has already been stated in this SOCIETY by Dr. Wm. H. Byford, we can, by thoroughly stretching the sphincter of the anus, get into almost any abscess which is truly pelvic in its nature, and points, or opens, in the lower four or five inches of the rectum. We can dilate the opening, and then enlarge it with the fingers, without the use of any cutting instruments, against which objection has just been made. The danger of hæmorrhage from the wounding of blood-vessels is certainly very much less than the dangers in operating by laparotomy. There must be danger connected with any operation, but in the one I am speaking of it is reduced to the minimum. The entrance of fæces is of minor importance, for in the presence of perfect drainage and antiseptic irrigations, they do not remain in the cavity, and do not prevent it from filling in with granulations from the top and sides, and contracting and healing. Therefore dilatation *per rectum* should be first tried; and if any cases, after trial, cannot be thus successfully treated, laparotomy, or some other substitute, should be considered. Professor Fenger quotes, and then criticises, my arguments against laparotomy, without mentioning that I was only referring to abscesses that could be reached through the rectum. With regard to recto-abdominal fistulæ, I must still maintain that the abscess will close up and get well more readily with one opening into the rectum, properly made or enlarged, than if such a fistula exist in which the rectal outlet has not been enlarged. Gases and fæces will almost invariably pass through and keep the abdominal end of the fistula open, and will lodge in septic pockets, or sinuses, resulting from the inadequacy of the opening at the rectal end. If we close such a fistula above, we have the unfavorable conditions of the original abscess, and must still practice dilatation per rectum. Martin's method of instituting perfect drainage through the vagina, and then closing the abdominal opening, would undoubtedly be sound in principle, were it not that an operation through the vagina should take precedence when practicable; and when not, a single opening from above must be regarded as fulfilling the requirements, as is proved by the experience of Tait and others. Hence Martin's method must eventually be relegated to the exceptional procedures.

I do not wish the SOCIETY to understand that I do not believe in laparotomy for pelvic abscess, but that, being popular and new, it is apt to be resorted to unnecessarily and unjustifiably. The remarkable success of Tait should not be allowed to mislead us. His mastery of *technique* and fertility of resource, in abdominal surgery, justifies him in assuming risks which others may not.

PROFESSOR EDMUND ANDREWS: It seems to me that the usefulness of the operation in proper cases is reasonably certain, but that we must be careful not to incur the risks of laparotomy when safer methods are available.

It will broaden our views somewhat, if we recollect that an abscess in the cellular tissue about the uterus does not differ materially in nature, principles of treatment, nor results, from abscesses in the cellular tissue in other parts of the pelvis and abdomen. One set of laws governs them all, both in pathology and treatment. A point to be remembered is, that many cases recover spontaneously in early stages, contrary to the statements of some eminent men. A professional gynæcologist or surgeon, whose patients are attracted to him from long distances on account of his reputation, gets a class of cases, because of their long standing and obstinate character. He has to combat the same tendency to error of judgment in one respect which besets the mind of a pathologist, whose conclusions are too exclusively drawn from the dead-house; that is to say, neither of them sees the numerous cases which recover under ordinary treatment, and therefore do not come before them. Recoveries from abscesses in the cellular tissue in all parts of the abdomen and pelvis are common, though they are apt to be very slow.

I recall two cases, attended by well-known physicians in this city, which recovered from such abscesses after suffering about a year. These abscesses discharged through the rectum. I have, perhaps, been somewhat slow in the treatment of these cases. For example, a retrouterine abscess was brought to me from a distant state. It periodically discharged into the rectum above the reach of the finger. The patient arrived in Chicago in fair general health. Directly after her arrival, the discharges grew smaller in quantity, with longer intervals of time between, and she continued to progress in that way. Improving constantly in strength and activity, she began to make excursions and long visits to friends in neighboring states, and, in short, enjoyed life so thoroughly that I deemed a laparotomy not justifiable so long as she progressed so well towards recovery without it. I presume she got tired of my dilatory plan. At any rate, after some months of improvement she ceased to report herself for periodic examination, and I lost sight of the case. I have a case now on hand in a more debilitated condition. She is confined to her bed the greater part of the time. I expected to operate many weeks ago, but soon after I took charge of the case she showed signs of improvement, which led me to postpone the laparotomy to see what would occur. The discharges, which were from a point high up in the rectum, grew less in quantity and further apart in time. The temperature went down to the normal standard and remained there, the tumor about the uterus diminished in size, and nearly two months have now elapsed since the last small discharge of pus. The patient's vigor is slowly returning. Under such conditions it is not certain that any pus cavity remains. I deem it my duty to wait until the presence of such a collection of pus is reasonably certain before subjecting the patient to the perils of laparotomy. Not long ago I had the opportunity to make a post-mortem examination in a case of circumuterine abscess. The abscess had formed several years ago, after a difficult parturition. I saw the patient in consultation a few times during the last weeks of life. The pus was discharged, partly by the rectum and partly through a fistula midway between the *symphysis pubis* and the umbilicus. She had been subjected twice to some surgical operation, whose exact nature I did not learn. The operations were not laparotomies. Having received no benefit, the patient was determined that no more surgery should be tried on her. After some weeks she died of asthenia, and I was allowed a limited autopsy. The fistula above the pubes, after passing through the integuments, led downward and to the right, and at a point which seemed to be the right external inguinal ring it entered the inguinal canal and followed the round ligament into the pelvis. Here it became more spacious, but exceedingly crooked and complicated, winding irregularly backward until it opened into the upper part of the rectum. All the viscera in the vicinity were glued together in a mass by old inflammatory deposits. There was no large abscess cavity at any one point, but still there was a flattened pocket some three inches long, and an inch or more in width, lying behind the rectum in the hollow of the sacrum. It contained a little pus and fæces. This pocket might have been safely opened from below, working up outside the posterior surface of the rectum, had it been possible to ascertain its existence. I do not see how it could have been reached by laparotomy. The anterior fistula might have been benefited by freely slitting up the inguinal canal. I feel compelled to differ with my friend Dr. Henry T. Byford in one point. He suggests very naturally that Tait's operation, performed after the abscess cavity has opened into the rectum, would make a complete intestinal fistula, which it might be impossible to heal. This thought is natural, and I confess I would think the same thing myself, had not an extensive observation upon fæcal fistulæ shown me the reverse. Experience teaches that an abscess cavity opening into an intestine and filled with putrid pus and fæces, is very reluctant to heal so long as it is not freely drained and disinfected; but if it is widely opened, so as to make and maintain the shortest and straightest possible route from the opening in the cut to the external air, and if it be kept well cleansed and disinfected, fresh granulations will spring up, the orifice will contract and the fistula will heal, provided there is no stricture in the intes tine below. This fact, or law, is very important, and applies equally to fæcal and urinary fistulæ, as I have verified by an abundant experience. A striking case in point occurs to me at this moment. An eminent physician on the South Side requested me to take charge of one of his patients, a lady who seemed to have an anomalous hernia, and was sinking under a suppurative discharge from the bowels. On examination I found her confined to bed, and rapidly approaching fatal exhaustion. There were several evacuations daily of mingled pus and fæces from the rectum. The left hip was found prominent over the whole gluteal region. The tumor fluctuated on palpation, was resonant on percussion, and gave a succussion on coughing. At times it gurgled under pressure. I opened it very slowly and carefully, fearing to find an intestine there. After passing through the atrophied *gluteus maximus*, I entered a broad cavity containing neither intestine nor omentum, but filled with pus and fæces. This cavity being emptied and washed out, was easily traced up to the sciatic notch, where it entered the pelvis by an orifice of moderate size. I now ripped the cavity open for nearly its whole length, and kept it cleansed. Vigorous granulations sprang up at once and the sac healed up rapidly and permanently. The patient seemed relieved of a great depressing influence, and rebounded at once toward health. She became plump and rosy, and rapidly regained her full strength.

II. PROFESSOR F. E. WAXHAM presented for examination a

### FEEDING BOTTLE FOR USE IN CASES OF INTUBATION OF THE LARYNX.

The feeding bottle consists of an ordinary nursing flask, with a rubber cork, with a small vent through which a tube passes to the bottom of the bottle. To this tube is attached another leading to the bulb of a Davidson's syringe, and this in turn is attached to a small-sized œsophageal tube. In using this apparatus the gag is placed between the jaws, the tube introduced into the œsophagus, and the contents of the bottle quickly introduced by means of the bulb.

Many patients, especially young infants, do not take sufficient nourishment after intubation has been performed, on account of the coughing produced by the trickling of the liquid into the trachea. This apparatus obviates the difficulty.

W. W. JAGGARD, M.D., *Editor.*

2330 Indiana avenue, 15th March, 1886.

